# Functional Comparison of XPF Missense Mutations Associated to Multiple DNA Repair Disorders

**DOI:** 10.3390/genes10010060

**Published:** 2019-01-17

**Authors:** Maria Marín, María José Ramírez, Miriam Aza Carmona, Nan Jia, Tomoo Ogi, Massimo Bogliolo, Jordi Surrallés

**Affiliations:** 1 Departament de Genètica i de Microbiologia, Universitat Autònoma de Barcelona, 08028 Barcelona, Spain; mariamarinvilar@gmail.com (M.M.); mariajose.ramirez@uab.cat (M.J.R.); miriam.azacarmona@gmail.com (M.A.C.); 2 Centro de Investigación Biomédica en Red de Enfermedades Raras (CIBERER), 08028 Barcelona, Spain; 3 Institute of Medical and Molecular Genetics (INGEMM), Hospital Universitario La Paz, 28029 Madrid, Spain; 4 CIBERER, ISCIII, 28029 Madrid, Spain; 5 Department of Genetics, Research Institute of Environmental Medicine (RIeM), Nagoya University, Nagoya, Japan/Department of Human Genetics and Molecular Biology, Graduate School of Medicine, Nagoya University, Nagoya 464-0805, Japan; jia.nan@riem.nagoya-u.ac.jp (N.J.); togi@riem.nagoya-u.ac.jp (T.O.); 6 Genetics Department Institute of Biomedical Research, Hospital de la Santa Creu i Sant Pau, 08025 Barcelona, Spain

**Keywords:** XPF-KO, *XPF*/*ERCC4* mutations, DNA repair, genotype-phenotype correlation

## Abstract

XPF endonuclease is one of the most important DNA repair proteins. Encoded by *XPF*/*ERCC4*, XPF provides the enzymatic activity of XPF-ERCC1 heterodimer, an endonuclease that incises at the 5’ side of various DNA lesions. XPF is essential for nucleotide excision repair (NER) and interstrand crosslink repair (ICLR). *XPF*/*ERCC4* mutations are associated with several human diseases: Xeroderma Pigmentosum (XP), Segmental Progeria (XFE), Fanconi Anemia (FA), Cockayne Syndrome (CS), and XP/CS combined disease (XPCSCD). Most affected individuals are compound heterozygotes for *XPF*/*ERCC4* mutations complicating the identification of genotype/phenotype correlations. We report a detailed overview of NER and ICLR functional studies in human XPF-KO (knock-out) isogenic cells expressing six disease-specific pathogenic XPF amino acid substitution mutations. Ultraviolet (UV) sensitivity and unscheduled DNA synthesis (UDS) assays provide the most reliable information to discern mutations associated with ICLR impairment from mutations related to NER deficiency, whereas recovery of RNA synthesis (RRS) assays results hint to a possible role of XPF in resolving R-loops. Our functional studies demonstrate that a defined cellular phenotype cannot be easily correlated to each XPF mutation. Substituted positions along XPF sequences are not predictive of cellular phenotype nor reflect a particular disease. Therefore, in addition to mutation type, allelic interactions, protein stability and intracellular distribution of mutant proteins may also contribute to alter DNA repair pathways balance leading to clinically distinct disorders.

## 1. Introduction

**Figure 1:**
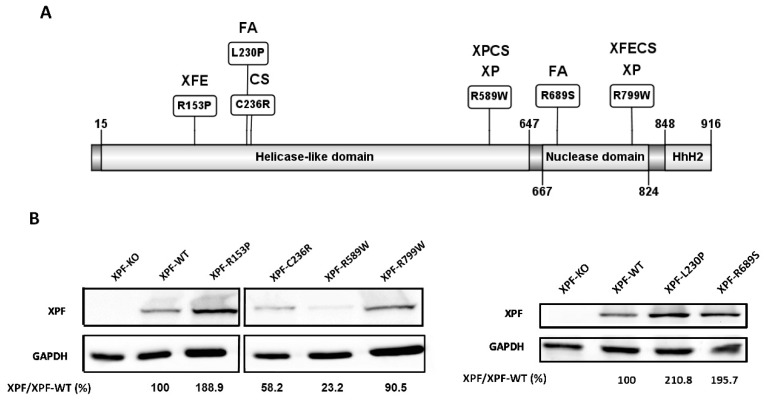
(**A**) Schematic view of the XPF domains with the selected mutations and the diseases found in patients. The patient XPCSCD had an allele with C236R and the other with R589W. R799W mutation found in homozygosis in XP was also found in a cohort of patients with XFE sharing CS features. This figure was created with a protein designing software from [44]. XP: Xeroderma Pigmentosum; CS: Cockayne Syndrome; XPCSCD: Xeroderma Pigmentosum Cockayne Syndrome combined disease; XFE: Segmental Progeria. (**B**) Western Blot (WB) levels of each single XPF mutant transduced cell line. The two WB are split by spatial reasons. Levels of XPF proteins are normalized to GAPDH expression levels and expressed as a percentage relative to the exogenous XPF-Wt. GAPDH is used as a loading control.

The human *XPF*/*ERCC4* gene is located in 16p13.1-p13.2 and encodes for the 916 amino acids long XPF protein, [1] which forms a stable heterodimer with ERCC1 in order to constitute a structure-specific endonuclease that incises the 5’ side of several types of DNA lesions. XPF-ERCC1 heterodimer is essential for normal development, since the complete inactivation of the *XPF*/*ERCC4* or *ERCC1* in humans and mice is incompatible with postnatal survival [2,3,4,5]. XPF is organized in three different domains (Figure 1A): an N-terminal helicase domain (residues 15–647), a central nuclease domain (residues 667–824) and a C-terminal helix-hairpin-helix (HhH) domain (residues 848–916) [6]. XPF specifically recognizes single strand DNA (ssDNA) through its N-terminal helicase domain, while ERCC1 binds to double-strand DNA (dsDNA) through its hairpin region. This different substrate specificity allows the heterodimer to bind to both single and double-strand DNA and determines the incision position during DNA repair [7]. *XPF*/*ERCC4* was originally identified as the defective gene in xeroderma pigmentosum complementation group F (XP-F) [8] since wild type *XPF*/*ERCC4* cDNA complemented human XP-F cells as well as nucleotide-excision repair (NER) deficient *Ercc4* and *Ercc11* rodent cells [9]. XPF-ERCC1 dimer is able to establish temporal interactions with other proteins such as XPA, RPA and SLX4 to participate in several damage repair pathways [10,11,12,13]. The XPF-ERCC1 heterodimer participates in NER [8], interstrand crosslinks repair (ICLR) [14], microhomology-mediated end joining (MMEJ) [15], single strand annealing (SSA), a branch of double-strand breaks (DSB) repair, [15] and telomere maintenance [16]. Moreover, XPF-ERCC1 has been found to have possible backup roles in repairing oxidative damage and DNA breaks with damaged ends [17]. Additionally, it has emerged as a potential target for inhibitors to sensitize cancer cells to DNA damage-based chemotherapy [18,19]. Considering its wide involvement in DNA repair machinery, it is not surprising the range of human diseases associated with mutations in the *XPF*/*ERCC4* and *ERCC1* genes.

Nucleotide-excision repair is one of the most versatile DNA damage repair pathways. It is involved in the removal of lesions caused by ultraviolet (UV) radiation like cyclobutane-pyrimidine dimers (CPDs) and 6–4 photoproducts (6–4 PPs), several natural and induced bulky chemical adducts, intrastrand crosslinks and ROS-generated cyclopurines. Many of these lesions distort the DNA double helix and must be removed to allow proper DNA replication and transcription [20]. NER is divided in two subpathways: global genome repair (GGR) and transcription-coupled repair (TCR), which differ in the damage recognition process but share the same mechanism to incise at both sides of the lesion, repair and ligate the DNA gap. In the GGR subpathway the entire genome is probed by the protein sensor XPC to check for damage, while TCR is activated when UV-induced lesions arrest the transcriptional machinery. When XPC recognizes the damage or RNA pol II stalls at the damage site, the transcription initiation factor IIH (TFHII) is recruited and XPD and XPB open the double helix. XPA verifies the damage and RPA occupies the non-damaged strand and help to recruit XPF-ERCC1 heterodimer to incise at 5′ of the damage. XPG then incises the 3′ side of the lesion producing a gap in the damaged strand of 22–30 nucleotides that is filled and sealed by DNA polymerases and ligase activities [20].

Several genome instability syndromes may arise if NER is compromised. One of them is xeroderma pigmentosum (XP; OMIM 278760), an autosomal recessive syndrome with 100% penetrance characterized by extreme photosensitivity and a 10,000-fold increased risk of skin cancers due to failure to repair DNA lesions produced by UV light [21]. Ocular abnormalities and increased risk of cancers of the oral cavity are also very common among the patients [22]. Eight XP complementation groups (XP-A to XP-G and XP-V) are described: proteins from XPA to XPG are involved in the repair of the UV lesions while XPV (pol η) is involved in bypassing DNA lesions by translesion DNA synthesis above the damaged nucleotides. Most XP-F patients have mild XP symptoms and a reduced level of nuclear XPF protein since most mutations promote XPF-ERCC1 mislocalization to the cytoplasm of cells and lead to insufficient levels of XPF-ERCC1 to complete NER [23]. 

Similar to *XPD*, which mutations can cause up to six different clinical phenotypes [24], *XPF*/*ERCC4* mutations give raise to diseases other than XP. Mutation in *XPF*/*ERCC4* can produce a progeria-like phenotype (XFE) characterized by the failure of the mutant XPF protein to properly translocate to the nucleus and to be recruited to sites of active DNA repair. The patients present severe photosensitivity, neurological and musculoskeletal abnormalities and hematopoietic symptoms [25,26]. *XPF*/*ERCC4* mutations can produce Cockayne syndrome (CS), an autosomal recessive disorder associated with a defective TCR [27,28]. CS patients show neurological and developmental abnormalities, growth and mental retardation, microcephaly, premature ageing and abnormal skin photosensitivity but that does not lead to skin cancer [29]. Some other rare *XPF*/*ERCC4* variants can produce in the patients combined features of CS and XP. These cases present severe UV sensitivity and cancer predisposition typical of XP and developmental abnormalities which are common in CS patients [27,30].

One of the most dangerous DNA lesions are interstrand crosslinks (ICLs), since they block DNA strands separation thus inhibiting DNA replication, transcription and segregation [31]. ICLs can be produced as a result of cellular metabolism or by chemotherapeutic drugs such as mitomycin C (MMC), diepoxybutane (DEB), cisplatin, nitrogen mustard and psoralens [31]. The detection and repair of these lesions require a strict organization of multiple DNA repair proteins organized in the Fanconi anemia (FA)/Breast cancer (BRCA) DNA repair pathway [32]. Mutations in at least 22 genes involved in the FA/BRCA pathway cause FA, a rare genetic disease with an incidence of 1–9 in 1,000,000 live births and an estimated carrier frequency of 1 in 250 in most populations. Its clinical features include bone marrow failure, pancytopenia, hyperpigmentation, skeletal malformations, small stature and urogenital abnormalities and predisposition to leukemias and solid tumors. FA cellular phenotype is characterized by extreme sensitivity to DNA cross-linking agents and chromosomal fragility [32,33].

FA proteins can be divided in three functional groups: the FANCore complex, the ID complex and the downstream proteins [32]. The FANCore complex is formed by seven FA proteins and six FA-associated proteins whose functions are to activate through monoubiquitination the heterodimer formed by FANCD2 and FANCI proteins (ID complex) to enable its relocation to the DNA damage site [34] where it allows the recruitment of SLX4-XPF-ERCC1 to incise and unhook the ICLs. Despite several other endonucleases being involved in ICLSs processing [12], the identification of FA patients carrying *XPF/ERCC4* mutations suggests that XPF-ERCC1 endonuclease has a major role in ICL repair [35]. In accordance with these observations, in vitro models [6,36] and mice models [11,37] confirm XPF as the main endonuclease involved in the physiological unhook of ICLs.

All *XPF/ERCC4*-mutated patients regardless their clinical phenotypes carry at least one allele with a missense mutation that does not affect dramatically its catalytic domain [21,27,35]. Taken together with the observation that mice homozygous for *Ercc4* or *Ercc1* null alleles are not viable, these data suggest that XPF-ERCC1 activity is essential for life.

Geneticists have identified several missense mutations in *XPF*/*ERCC4* associated with a distinct clinical phenotype (Figure 1A). Variant c.458G>C, p.Arg153Pro, was found in homozygosity in an XFE patient [25]. This amino acid change affects the helicase domain, a leucine-rich region involved in the interaction with SLX4 and DNA binding [6]. The patient’s main feature was the accelerated ageing phenotype and the disease was named as XFE progeroid syndrome [25]. Mutation c.689T>C, p.Leu230Pro was found in the paternal allele of an FA patient carrying a truncated maternal allele [35]. Mutation c.2065C>A, p.Arg689Ser was found in another FA patient along with another truncated allele and it was proven to affect XPF excision activity [35]. Variant c.706T>C, p.Cys236Arg was found in heterozygosity in two different patients: along with a frameshift mutation in the other allele caused CS [27], while in heterozygosity with the missense mutation c.1765C>T, p.Arg589Trp produced a CS/XP combined syndrome [27]. The c.1765C>T, p.Arg589Trp variant was previously shown to be associated with different XP clinical phenotypes: combined with a deletion in exon 3 of the second allele caused severe XP, while in heterozygosity with the missense mutation p.Arg799Trp caused mild XP [23,28,38]. The c.2395C>T, p.Arg799Trp missense mutation was found in homozygosis in a patient diagnosed with mild XP [39], but it was recently found along with a truncated allele in a patient with progeria syndrome [26].

Concerning the wide range of diseases associated with changes in XPF protein due to its crucial role in several DNA repair pathways, the objective of this study was to better understand the role of XPF in DNA repair and human disease by analyzing the genotype-phenotype correlation of *XPF*/*ERCC4* pathogenic variants causing XP, FA, CS, XFE or CS/XP in a genetically homogeneous background.

## 2. Materials and Methods

### 2.1. Cell Culture

All the cell lines used in this study were cultured in DMEM (Dulbecco’s Modified Eagle Medium, Biowest cat. no. L0104, Barcelona, Spain) complemented with 10% FBS (Fetal Bovine Serum, Biowest cat. no. S181B) and 0.1 μg/mL Plasmocin (IBIAN Technologies, Zaragoza, Spain). Cells were cultured at a controlled temperature of 37 °C in 5% CO_2_ atmosphere.

### 2.2. Generation of XPF-KO CELL Line

XPF-KO cell line was generated by TALEN targeting *ERCC4* exon 2 sequence: 5′ TCGCCGTGTAACAAATGAAATCACAAGCAACAGTCGCTATGAAGTTTACACA3′ (Underlined fragment represents TAL binding sites; central region represents FokI endonuclease cutting site) in HEK 293T (Human Embryonic Kidney 293T cells, ATCC CRL-11268). A reporter plasmid [40] containing the same *ERCC4* recognition sequence, a red fluorescent protein sequence and an out of frame green fluorescent protein (GFP) that could be restored when the DSB produced by FokI was repaired by nonhomologous end joining (NHEJ) was also used. Then, 48 h post transfection double fluorescent (red and green) cells were selected by flow cytometry using the FACSAria II (BD Bioscience). Single cell cloning of these cells was performed by limit dilution in three 96-well plates. After 2 to 3 weeks, three individual clones were picked to check the lack of XPF protein by Western Blot (WB).

### 2.3. Western Blot Analysis

A total of 1 × 10^6^ of cells were lysed in 50 μL of RIPA 1x lysis buffer (Millipore 20–188, Wien, Austria) with Benzonase nuclease (10 U/mL final) (VWR International 7074-6-3, Llinars del Vallés, Spain). Samples were incubated at 37 °C for 10 min and then centrifuged at 13,000 rpm. Total protein concentration of the supernatant was determined by Bio-Rad Protein Assay (Biorad, Hercules, CA, USA) according to manufacturer’s instructions and 50 μg of total proteins were denaturalized for 10 min at 96 °C in Laemmli 1x (Sigma S3401-10VL, Saint Louis, MO, USA) and loaded in an 8% SDS-PAGE. Proteins were transferred to nitrocellulose membrane with the iBLOT2 (Invitrogen, Thermo Fisher Scientific, Waltham, MA, USA) apparatus following manufacturer’s guidelines. Membranes were blocked in 5% of milk in TTBS for 1 h at RT, and immunodetection was performed by incubating the membranes with diluted primary antibodies in blocking solution for 16 h at 4 °C. Primary antibodies used were: anti-XPF (mouse monoclonal, Ab-1 219 Thermo Fisher Scientific) 1:200 and anti-Actin (rabbit polyclonal, ab1801, Abcam, Cambridge, UK) 1:1000. The following day, membranes were incubated in secondary antibodies conjugated to peroxidase during 1 h at RT and revealed with Pierce ECL Western Blotting Substrate (Pierce). Digital images of the membranes were captured with a GeneGnome apparatus (Syngene Bio imaging, Bangalore, India). GeneTools analysis software (Syngene Bio imaging) was used to quantify the amount of protein per band.

### 2.4. Characterization of the Selected XPF-KO Clone

Genomic DNA was extracted from 5 × 10^6^ clonal XPF-KO cells using the DNeasy Blood & Tissue Kit (Qiagen, Hilden, Germany) according to manufacturer’s instructions and DNA concentration was measured by spectrophotometry using NanoDrop ND-1000 (NanoDrop Technologies). PCR to identify mutations inserted by TALEN was performed using the following primers: For (TGTAGACTGGTTGGCTGAAGT) and Rev (CGCCTATGTGCTTCCCAAGA). DNA was amplified by denaturation at 94 °C for 30 s, annealing at 58 °C for 30 s, and elongation at 72 °C for 4 min for 35 cycles. Subsequently, the target DNA was elongated at 72 ºC for 4 min. The product size was checked into a 1.5% agarose gel, the DNA band was purified and subcloned by TOPO TA cloning (Thermo Fisher Scientific), transformed in One Shot TOP10 Chemically Competent *Escherichia coli* cells (Life Technologies, Carlsbad, CA, USA), amplified by performing Minipreps with NucleoSpin Plasmid (Macherey-Nagel, Düren, Germany) and sent to Sanger sequencing at Macrogen Inc. (Seoul, Korea).

### 2.5. Generation of the XPF Mutant Variants

HA tagged *ERCC4* cDNA [35] was subcloned into a 3rd generation lentiviral vector pULTRA (a gift from Malcolm Moore (Addgene plasmid # 24129)) downstream of the EGFP-P2A site, to produce a bi-cistronic expression of EGFP and XPF. The ligation product was then transformed using One Shot Stbl3 Chemically competent *E. coli* (Thermo Fisher Scientific), amplified by standard Miniprep and Maxiprep procedures and sent to Sanger sequencing of Macrogen.

Single nucleotide variants generated by site directed mutagenesis were: c.458G>C, p.(Arg153Pro); c.689T>C, p.(Leu230Pro); c.706T>C, p.(Cys236Arg); c.1765C>T p.(Arg589Trp); c.2065C>A, p.(Arg689Ser); and c.2395C>T, p.(Arg799Trp). The QuickChange II XL Site-Directed Mutagenesis Kit (Agilent Technologies) was used following manufacturers’ instructions to design mutagenic primers (https://www.genomics.agilent.com/primerDesignProgram.jsp) (Supplementary Table S1) to introduce the variants in the wild type (wt) cDNA of XPF. After PCR amplification, DpnI restricition enzyme was added for 1 h at 37 °C and the PCR product was transformed by XL10-Gold Ultracompetent Cells (Agilent Technologies), plated in LB Agar with Ampicilin (100 μg/mL). DNA from single colony minipreps was controlled by Sanger sequencing (Macrogen).

### 2.6. Generation of the XPF Lentiviral Particles and Genetic Complementation of the XPF-KO Cells

Production of lentiviral particle was achieved as in [41]. A total of 180,000 XPF-KO cells were seeded in a 12-well plate, then, 24 h later, cells were infected with 40 μL of each lentiviral particles and 1.5 μL Polybrene (Sigma). Three days after the infection, green fluorescence was detectable and cells that had integrated the virus with the XPF cDNA were selected by flow cytometry using the FACSAria II (BD Bioscience).

### 2.7. Ultraviolet C Sensitivity Survival Assay

XPF-KO cells expressing the different XPF variants were seeded in 2 mL of complete medium per duplicate in a 6-well plate. The, 24 h afterwards, medium was removed, cells were washed with PBS and UVC irradiated (254 nm; 15 W UVC Lamp G15-T18 Philips) at the doses of 0, 2, 5, 10 and 15 J/m^2^. Complete medium was then added, and survival cells were counted after 72 h with a Beckman-Coulter Cell Counter. At least three independent assays were performed, and results were expressed as a percentage of irradiated viable cells versus (vs) viability of the untreated controls.

### 2.8. Unscheduled DNA Synthesis Assay

A mix of poly-L and poly-D Lysine (Sigma-Aldrich) was used to coat 96-well plastic plates to improve cell attachment. A total of 8 × 10^4^ cells of each transduced cell line were seeded in 100 μL of complete medium, seeding 10 replicate wells per cell line, from which half of them were UVC irradiated (254 nm) at 20 J/m^2^ after 16 h, while the other half remained as non-treated controls. After UV irradiation, cells were incubated in DMEM without FBS with 5-ethynyl-2′-deoxyuridine (EdU) (Invitrogen). After 2 h of incubation at 37 °C, cells were fixed in 100 μL of Fixation Buffer (300 mM Sucrose, 2% Formalin, 0.5% Triton X-100 and PBS) and incubated for 20 min on ice. EdU was detected by adding 41 μL/well of EdU Detection Solution (50 mM Tris-HCl pH 7.3, 4 mM CuSO4, 10 mM Sodium Ascorbate, 10 μM Alexa 488-conjugated azide, 20 ng/mL DAPI and H_2_O) for 1 h. Then, cells were washed in PBS 0.05% Tween-20 for 40 min, fixed with 100 μL Formalin/PBS (1:10) for 20 min and image acquisition and data processing achieved using a high content screening (HCS) system, the ARRAY SCAN VTI (Thermo Fisher Scientific). Plates were scanned with a CCD camera-equipped fluorescence microscope and the images were processed with the software Cellomics Scan (Thermo Fisher Scientific). At least three independent UDS assays were performed and results are represented as fluorescence intensity of treated and non-treated cells.

### 2.9. Recovery of RNA Synthesis Assay

Coating and seeding of the cells were performed as for the UDS assay. After 16 h, half of the cells were UVC irradiated (20 J/m^2^) and incubated for 8 h at 37 °C in DMEM with 1% FBS serum (to allow recovery of RNA synthesis) before incubation during 2 h at 37 °C in 60 μL/well of serum-free DMEM supplemented with 5-ethynyluridine (EU) (100 μM). Detection and image acquisition were done as previously described. Detailed UDS and RRS methodologies can be found in [42].

### 2.10. Diepoxybutinate Sensitivity Survival Assay

A total of 2 × 10^5^ cells of each transduced line were seeded in 2 mL of complete medium per duplicate in a 6-well plate. Then, 24 h afterwards, DEB was added at a final concentration of 0, 0.025, 0.05, 0.1 and 0.2 μg/mL. Cells were grown during the time needed by the untreated to perform at least three population doublings. Afterwards, cells were trypsinized and counted with a Beckman-Coulter Cell Counter. At least three independent assays were performed, and results are expressed as a percentage of treated viable cells vs the untreated controls.

### 2.11. Dieopxybutinate-Induced G2/M Cell Cycle Arrest

A total of 1 × 10^6^ cells of each cell line were seeded in 3 × 25 cm^2^ flasks (F25) in 5 mL of complete medium. Then, 24 h afterwards, DEB was added at doses of 0, 0.005, 0.01, 0.025 and 0.05 μg/mL. Then, 48 h after the treatment, cells were trypsinized, PBS washed and resuspended in 200 μL of PBS. 2 mL of ice-cold ethanol 70% was added and cells were placed for 30 min on ice. Ethanol was removed by centrifugation and substituted with 2 mL of Staining solution (40 μg/mL Propidium Iodide, PI, Invitrogen, Carlsbad, CA, USA; Pure Link RNasa A 0.1 mg/mL, Invitrogen; PBS 1x). Cell cycle population distribution depending on DNA amount was analyzed by flow cytometry with FACSCalibur (BD Biosciences, Allschwil, Switzerland) as in [35]. A total of 15,000 events were registered per sample and data was analyzed by FlowJo VX software.

### 2.12. Chromosome Fragility by the Flow Cytometric Micronucleus Test

Around 3 × 10^5^ cells from each cell line were seeded in 6-well plates. Then, 24 h later, they were untreated or treated with 0.01 μg/mL of DEB and kept in culture for enough time for at least one population doubling. Cells were then sequentially stained; first with ethidium monoazyde bromide (EMA) (0.025 mg/mL) and secondly with Sytox green (0.2 μM). EMA covalently binds to chromatin of dying and dead cells after a photo-activation step achieved by keeping cells under a 60 W light bulb (about 30 cm distance) for 20 min. Following this, cells were washed in cold PBS with 2% FBS. After that, a lysis step with 250 μL of lysis solution 1 (0.584 mg/mL NaCl, 1 mg/mL sodium citrate, 0.3 μg/mL IGEPAL, 1 mg/mL RNase A and 0.2 μM Sytox green in deionized water) for 1 h at RT was done. Later, a second lysis step was done by adding 250 μL of solution lysis 2 (85.6 mg/mL sucrose, 15 mg/mL citric acid and 0.2 μM Sytox green in deionized water) for 30 min at RT. After lysis, samples were stored at 4° C until being processed by flow cytometry (up to two days). Data acquisition was performed by flow cytometry with FACSCalibur; Sytox-associated fluorescence was detected by FL1 channel while EMA-associated fluorescence was detected by FL3 channel. Collected data was analyzed by Flow Jo VX software. The data of micronuclei (MN) presented in this work represents results from five independent experiments each one in duplicate.

## 3. Results and Discussion

TALEN technology was used to edit the genome of HEK 293T cells to generate an *XPF*/*ERCC4*^-/-^ human cell line (XPF-KO). The system included an RFP expressing plasmid (Surrogate plasmid) [40] with an out of frame GFP that could be restored when a DSB produced in the target recognition sequence in the *XPF*/*ERCC4* gene was repaired by NHEJ. If TALEN proteins were functional, cells showed red and green fluorescence (Supplementary Figure S1A). After cell sorting and single cell cloning, clone number one was selected by Western blotting (WB) and sequence analysis (Supplementary Figure S1, panels B and C). Genetic complementation of the cellular phenotype of UV sensitivity with wild type *XPF*/*ERCC4* cDNA definitely validated the XPF-KO clone (Supplementary Figure S1B,D). Genomic DNA analysis revealed that the mutations introduced by the TALEN in *XPF*/*ERCC4* were two different deletions of 4 and 7 bp respectively: c.281_284del and c.280_286del (Supplementary Figure S1C). Sequencing of the whole *XPF*/*ERCC4* confirmed that the rest of the sequence remained unaltered.

All the cDNAs of the different *XPF/ERCC4* variants were transduced in the same conditions in XPF-KO, and all the XPF variant proteins were detectable by WB (Figure 1B). XPF-R799W showed levels of expression similar to wild type XPF (XPF-WT). XPF-R153P, XPF-L230P and XPF-R689S showed increased levels of protein production compared to XPF-WT. This was in contrast to the reduced levels observed in patients [25,26,35,39] but at the same time these levels of expression ensured that the cellular phenotype-genotype correlation depends only on the mutation and not on protein’s quantity. However, XPF-C236R and XPF-R589W showed decreased levels in vitro, similar to the levels observed in the patients [27,43], indicating that these XPF variants are inherently unstable independently of expression conditions and genetic backgrounds.

**Figure 2:**
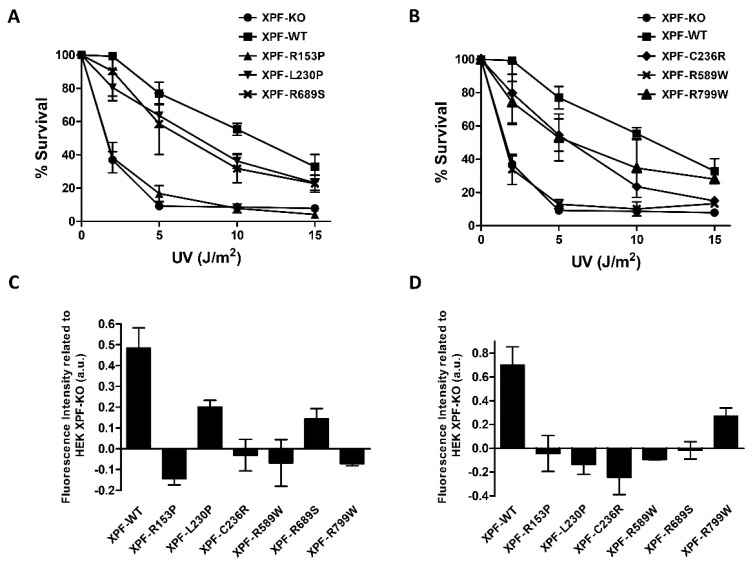
(**A**,**B**) Percentage of surviving cells after increasing doses of ultraviolet (UV) irradiation. Mean of at least two independent experiments of two replicates and the standard deviation is represented in the two graphs. (**C**) Unscheduled DNA synthesis (UDS) assay. Fluorescence intensity is represented relative to fluorescence intensity of Human Embryonic Kidney (HEK) XPF-KO. The graph represents the mean of at least three independent experiments with SD. (**D**) RRS assay. Fluorescence intensity is represented relative to fluorescence intensity of HEK XPF-KO. The graph represents the mean of at least three independent experiments with SD.

Cell lines expressing the different XPF variants were tested for sensitivity to UVC irradiation. For a better understanding, data were split into two different graphs: Figure 2A includes controls, XPF-R153P (XFE), XPF-L230P (FA) and XPF-R689S (FA) while the second graph (Figure 2B) shows controls, XPF-C236R (CS), XPF-R589W (CS/XP) and XPF-R799W (XP and XFE/CS). All the variants showed increased UVC sensitivity and cells expressing XPF-R153P and XPF-R589W were the most sensitive to UV, similar to XPF-KO (Figure 2A,B). These results are in concordance with the phenotype of the patients where these mutations were identified: XFE progeria patient showed skin photosensitivity and patient’s primary fibroblasts were 10 times more sensitive to UVC [25]. XPF-R589W was found in XP patients and in an XP/CS patient XPCS1CD together with XPF-C236R variant [21,28]. Interestingly, another patient (CS1USAU) with XPF-C236R together with a XPF null allele p.Tyr577* had only the CS phenotype [27]. This could indicate that expression of XPF-R589W is actively causing the XP phenotype in patient XPCS1CD. In line with this hypothesis is the observation that, in our model and in [28], cells uniquely expressing XPF-C236R have mild UVC sensitivity (Figure 2B). A study performed by Popp and colleagues classifies for the first time XPF-R589W as an FA mutation [43]. The patient from which it was identified was presented as an FA clinical phenotype, however, bone marrow failure, one of the crucial features to be classified as FA, was absent. Despite the atypical FA clinical phenotype, it was the first FA patient to develop skin photosensitivity. The second allele of XPF presented a novel splice site mutation (c.793-2A>G) which produced a premature termination of translation (p.Thr265Valfs*13), hence originating a null allele as happened with the previously reported FA XPF variants [35]. Contrary to Hashimoto group, who found the XPF-R589W mutation was abruptly affecting XPF structure and its SLX4 binding [45]. Popp and colleagues detected a residual proportion of XPF-R589W escaping from protein misfolding, able to reach the chromatin [43] as happened with the reported FA XPF variants. The two FA XPF variants, XPF-L230P and XPF-R689S, showed a marked resistance to UVC when compared to XPF-KO (Figure 2A). These results confirmed previous studies which proved that these two mutations do not severely impair XPF to participate in NER of UVC-induced DNA lesions [35]. Interestingly, XPF-R589W mutant studied by [43] had reduced UVC irradiation resistance, and showed higher UVC irradiation resistance levels than XPF-L230P [43]. This combination of findings supports the conceptual premise that there is a link between ICL and NER pathways and endorse the theory that mutations found in specific locations of the sequence should not be associated with discrete DNA repair pathways impairment.

XPF-R799W showed a mild sensitivity to UVC (Figure 2B). XPF-R799W was initially found in homozygosis in an XP patient with a mild phenotype (XP42RO) [39] but it was recently identified in heterozygosis with an early truncated XPF allele in a patient (CALIF1010) with CS and XFE features [26] and in heterozygosis with XPF-R589W in a patient (XP24BR) with XP and CS features [38]. Interestingly, XPF-R799W sensitivity to UVC is very similar to the sensitivity of the CS associated XPF-C236R variant (Figure 2B). This result provides an explanation to the XP phenotype of patient XP24BR [38] double mutant XPF-R589W/XPF-R799W: similar to patient XPCS1CD (see above), in patient XP24BR XPF-R799W allele would be responsible for the patient’s CS features while the XPF-R589W allele would be responsible for the XP phenotype. These data also suggest a gene dosage dependent behavior for XPF-R799W: in homozygosis is associated to mild XP [39] while in heterozygosis with an allele with a deeply affected NER function it would be associated to atypical XFE and/or CS [26,38]. These data highlight the importance of mutated XPF alleles interactions to explain the variations of patients’ phenotypes.

To further discern among XPF variants, functional analysis of GG-NER and TC-NER were performed with the whole set of variants. Figure 2C shows UDS assay data representing the DNA repair ability by measuring DNA synthesis in the G1 phase of the cell cycle after DNA damage induced by UVC. This assay allowed a clear distinction from the XPF mutations that conferred UV sensitivity and the FA mutations. Despite this, all the variants showed a certain grade of impairment in UDS, the two FA associated variants, XPF-L230P and XPF-R689S, retained around 30% to 40% of their UDS activities when compared to the XPF-WT cells (Figure 2C). XPF-R153P showed the lowest UDS value and the CS and the XP associated variants, XPF-C236R, XPF-R589W and XPF-R799W, showed UDS levels similar to the XPF-KO. These observations are in line with the UDS levels of the patients-derived cells bearing these mutations [21,23,26,27]. These results demonstrate that XFE, XP or CS associated mutations impair NER much more then FA related mutations, and that expression of XPF-C236R, XPF-R589W, and XPF-R799W can be more impairing for UDS than the complete absence of XPF. These results are also indicative that UDS levels as UVC sensitivity could be used to distinguish FA associated XPF mutations from NER impairing XPF variants.

TC-NER capacity of our XPF mutants was evaluated by an RRS assay after UV induced damage: Figure 2D represents the ability of each cell line to synthetize RNA 8 h after UV damage in comparison with XPF-KO cells. All variants show a clear impairment of TC-NER including the FA associated XPF variants XPF-L230P and XPF-R689S. It has been observed that FA proteins, such as BRCA1, FANCD1, FANCD2, FANCA, and FANCM, are involved in resolving RNA-DNA hybrids known as R-loops. R-loops are structures formed when a nascent RNA hybridizes with the DNA template, leaving the non-template DNA single-stranded. R-loops are physiologically formed during transcription, but if they are not removed they can have deleterious effects on transcription, replication and genome integrity [46,47]. These loops are produced when a replication fork collides with a transcriptional nascent mRNA. Furthermore R-loops are present in telomeres and contribute to telomere maintenance [46,47]. XPF plays a role in telomere maintenance as it is known to be involved in the excision of T-loops [16] and it is responsible for telomeres loss when TRF2 is overexpressed [48]. According to the low RRS levels showed, a hypothesis to explain the low RRS sustained by the FA-associated variant is that XPF could be another FA protein involved in R-loops removal and expression of these pathogenic variants could result in RRS impairment. This would be strongly in concordance with the study of Sollier and colleagues [49], who provided evidence that XPF would be involved in resolving R-loops when knocking down some RNA processing factors such as AQR [49]. The R-loops are known to block replication forks and Fanconi proteins such as FANCD2 or FANCA are important to avoid their accumulations during S-Phase [46,47], and XPF as a component of the FA/BRCA DNA repair pathway could have an active role in this process. These observations also question RRS assay as a useful tool to discern between XPF mutations associated to different syndromes. It has been recently put up for debate if RRS levels should be used as a determinant feature for CS classification of the patients which has driven to an enlargement of diagnosis criteria focusing more in their clinical features [50].

**Figure 3:**
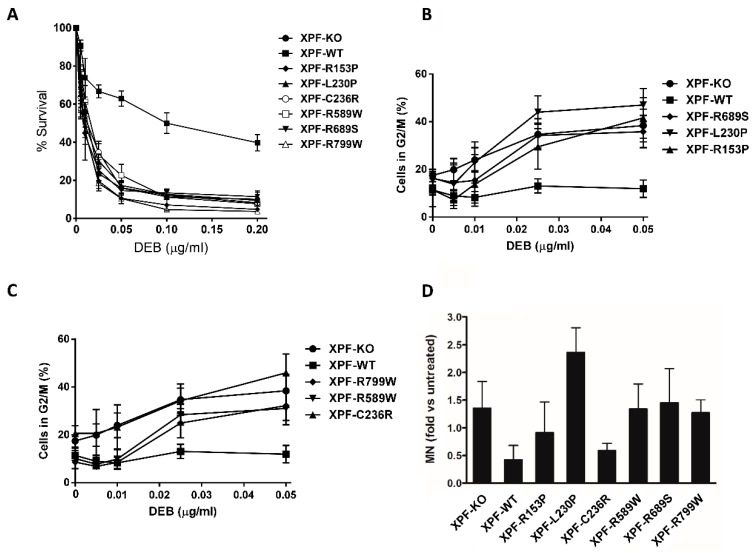
(**A**) Percentage of surviving cells under increasing doses of diepoxybutane (DEB). The graph represents the mean of at least two independent experiments with two replicates and the SEM. (**B**,**C**) Percentage of cells stalled in G2/M phase after DEB exposition. Half of the XPF variants are represented in each graph with the positive and negative controls. Each graph represents the mean of at least two independent experiments with the SEM. (**D**) Micronuclei (MN) test after DEB (0.01 μg/mL) exposure. Data is represented in fold changed vs. untreated cells. Graph represents the mean of at least three independent experiments with SD.

The repair of ICLs that covalently bind the two strands of DNA is crucial for the survival of cells. The role of XPF as the main endonuclease involved in the excision of the damage caused by these crosslinks is well supported [6,11,35,36,37]. To test our XPF variants in the repair of DNA crosslinks, a DEB survival test was performed and all the XPF variants expressing cells showed a marked ICL sensitivity, resembling the one of cells lacking XPF (Figure 3A). XFE Progeria associated variant XPF-R153P and XPF-R799W showed a strong sensitivity to DEB as already reported [25,26] such as the two FA associated mutants XPF-L230P and XPF-R689S [35] and the CS associated variant XPF-C236R, [6]. It has been shown that a replication-independent repair (RIR) of DNA interstrand crosslinks exists and that it works outside the S-phase. This system depends on both branches of NER and translesion synthesis polymerases [51] and defects in this mechanism are additive with the defects in ICLs repair during the S-phase [52]. One hypothesis to explain DEB sensitivity even of the XPF variants that are not causing FA, could be their inability to participate in RIR.

The accumulation of chromosome alterations caused by exposure to ICL agents is associated with a delay of the cell cycle to enter into mitotic phase. A DEB-induced G2/M cell cycle arrest assay was performed to test the different XPF variants. Again, for a better understanding of results, data have been plotted in two different graphs sharing the same controls (Figure 3B,C). All the XPF variants showed a higher percentage of G2/M arrested cells than the XPF-WT. The two FA associated mutations, XPF-L230P and XPF-R689S in concordance with previous studies [2,35] showed a marked G2/M block upon DEB treatment. Cells expressing progeria and CS associated mutation (XPF-R153P and XPF-C236R respectively) had very high levels of G2/M block, indicating that G2/M block after ICL treatment could be a useful additional tool for CS and XFE diagnosis. Regarding the remaining variants, XPF-R589W and XPF-R799W behaved as XPF-WT at the lowest DEB doses: XPF-R589W as expected from an XP associated variant would produce a more severe NER impairment than ICLR while XPF-R799W variant, associated to XP and XFE/CS features depending on the second allele, could have a minor impact on ICLR pathway when it is the only variant expressed in the cells. This will be in concordance with the observation, mentioned above, that when XPF-R799W is found in homozygosis patients suffer mild XP and not CS or FA. It would be interesting to generate cells expressing two different XPF mutated alleles to further our comprehension of genotype-phenotype interactions.

If left unrepaired, ICLs produce chromosome breaks that can result in MN detectable in daughter cells [53]. The flow cytometric MN test [54] provides useful information about the levels of chromosome breaks in a cell after ICL treatment by counting the number of MN in cycling cells (Figure 3D) by flow cytometry. The XPF-L230P expressing cell line showed the highest number of MN after DEB treatment, in concordance with the chromosome fragility phenotype of the FA patient in which it was identified [35]. The other FA associated mutation, XPF-R689S showed levels of MN similar to the XPF-KO (Figure 3D). XPF-R589W (XP) and XPF-R799W (XP, XFE/CS) variants also showed fragility levels resembling the ones of the XPF-KO cell line. Interestingly, R589W and R799W mutations showed high levels of MN, even if the percentages of G2/M arrested cells at this DEB dose were moderate (Figure 3C), indicating that the cells reach mitosis despite a heavy burden of chromosomal breaks thus implying a defect in the G2/M checkpoint. XPF-C236R, (CS), did not show chromosome fragility under DEB exposure, meaning that DNA damage is successfully resolved during the prolonged G2/M block (Figure 3C). The statistical error of XPF-R153P data was too large to reach any definitive conclusion.

The functional studies performed in isogenic human cell lines of several *XPF*/*ERCC4* missense variants enabled us to analyze if phenotypes are correlated with specific nucleotides changes or are influenced also by other factors. At first glance, it is remarkable how the position of the substitution along *XPF*/*ERCC4* sequence does not reflect the disease: as illustrated in Figure 1A, four variants were confined in the helicase-like domain and two in the nuclease domain, but the patients’ phenotypes were not determined by these positions. Regarding the analysis of XPF activity in the NER pathway, UV sensitivity and UDS assays provided the most reliable information to discern mutations more associated to ICLR impairment such as L230P and R689S (Figure 2A–C). The RRS assay, did not contribute significantly to discern among the XPF phenotypes (Figure 2D) however, the unexpected low RRS levels of FA associated XPF variants could imply XPF is another FA protein involved in the resolving R-loops. Further studies are needed to confirm this hypothesis.

Concerning ICLR analysis, there is a marked sensitivity of all variants and this combination of findings provides support for the conceptual premise that complete repair of ICLs requires S-phase dependent and S-phase independent DNA repair. Similar studies as the one performed in [52] with cells expressing the single XPF variants could be useful to find out if specific XPF mutations could be associated with one ICL DNA repair pathway or the other.

The message that stands out from our studies is that, despite the cellular functional analyses sometimes provide promising suggestions to understand the genotype/phenotype interactions (Supplementary Figure S2), a defined cellular phenotype cannot be correlated to each XPF mutation; functional analysis might help, but definitive statements about the contribution of XPF variants to the phenotype must take in account other factors such as XPF levels of expression, cellular localization, allelic interactions and the different genetic background of each patient.

## Supplementary Materials

The following are available online at http://www.mdpi.com/2073-4425/10/1/60/s1, Supplementary Figure S1: Generation and validation of the HEK XPF-KO clone, Supplementary Figure S2: Summary table of the whole set of XPF variants and their cellular phenotypes, Supplementary Table S1: Primers sequences used to introduce the exact single nucleotide variant of *XPF* by site directed mutagenesis.
