# Supplementary material for: Functional Comparison of XPF Missense Mutations Associated to Multiple DNA Repair Disorders
Source: Genes (Basel). 2019 Jan 17;10(1):60. doi: 10.3390/genes10010060 (PMC6357085; doi:10.3390/genes10010060)
Supplement: Supplementary file 1 [file genes-10-00060-s001.pdf]

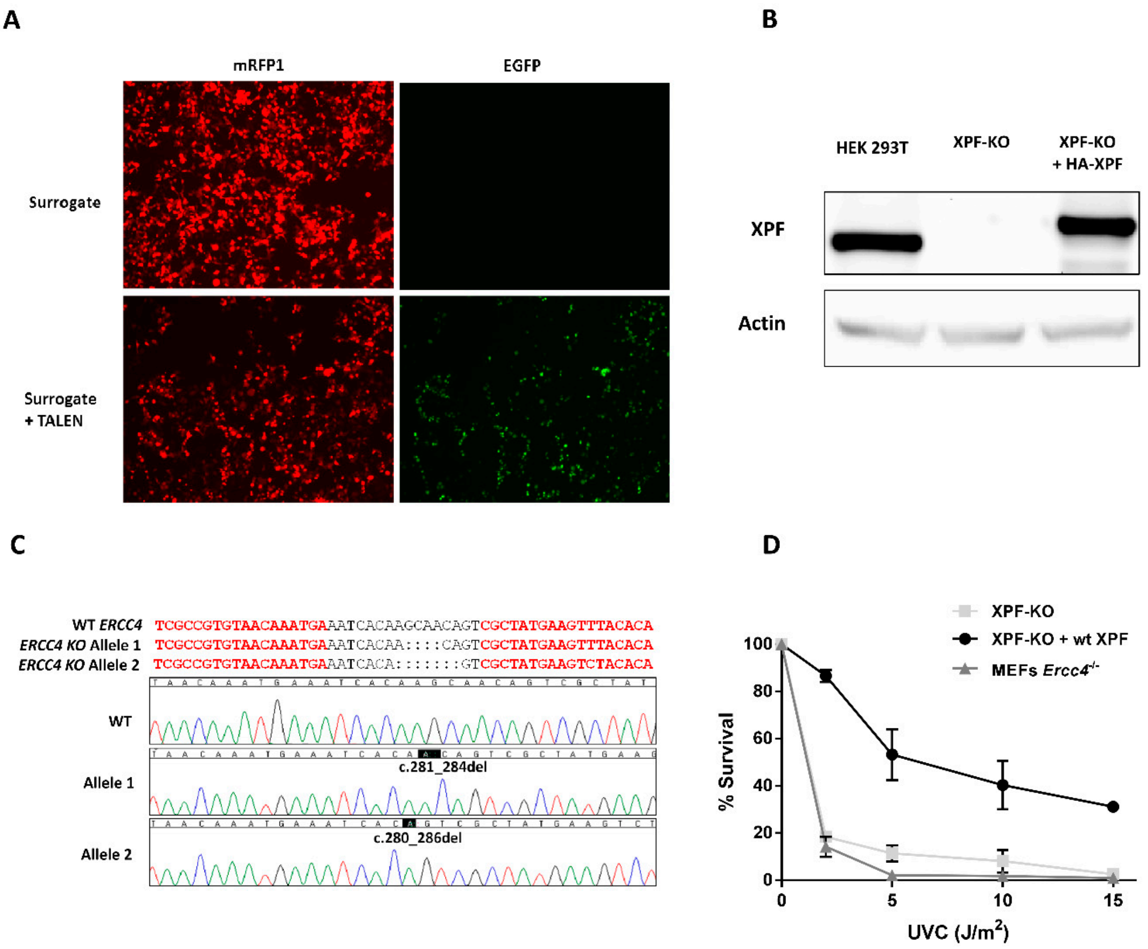

Supplementary Figure 1. Generation and validation of the HEK XPF-KO clone. (A) Analysis of TALEN endonuclease activity by fluorescence microscopy. Red fluorescence is detected in cells transfected with Surrogate (RFP+) vector; red and green fluorescence is detected in cells transfected with Surrogate + TALEN (RFP+GFP+) vector. No presence of double positive cells was found in cell cultures transfected just with the Surrogate vector. (B) WB validation of the HEK XPF-KO clone and the HEK XPF-KO transduced with the wt XPF. The slight difference in the molecular weight from the endogenous XPF of HEK 293T to the exogenous one was due to TAP and HA epitopes cloned in the ERCC4 sequence used for proteomic experiments (data not shown). Actin band is shown as a loading control. (C) Sequencing reaction chromatograms of heterozygous mutant clones. The KO clone was a compound heterozygote for a 4 bp deletion (c.281_284del) and a 7 bp deletion (c.289_286del) both in exon 2. (D) UVC cell survival to compare XPF-KO in different cell lines. HEK XPF-KO showed levels of UVC sensitivity comparable to MEFs Xpf-KO. Graph represents proliferation percentage media and SD of two independent experiments of two replicates.

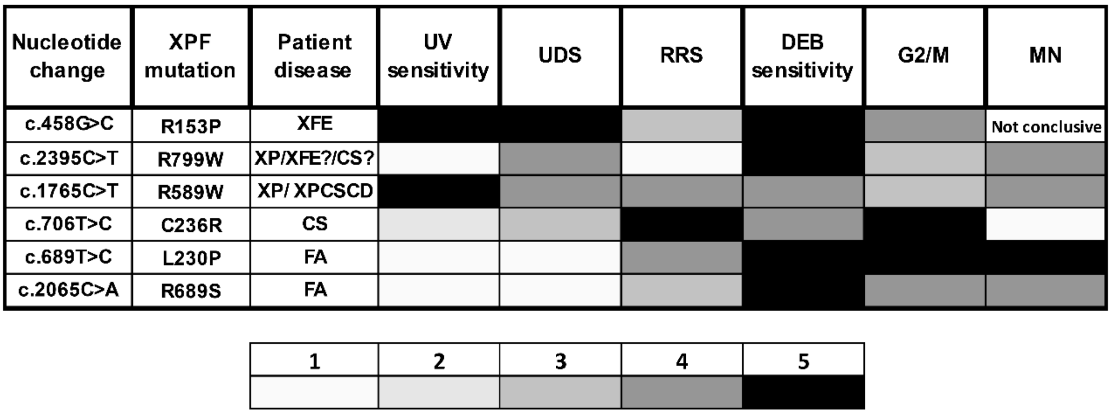

Supplementary Figure 2: Summary table of the whole set of XPF variants and their cellular phenotypes. For each ERCC4 nucleotide change, the XPF mutation and the pathology associated found in the patient where it was identified are described. The results of the functional assays performed with cells lacking XPF containing the variants are shown in a gray scale from 1 (less affected) to 5 (more affected).

Supplementary Table 1: Primers sequences used to introduce the exact single nucleotide variant of *XPF* by site directed mutagenesis.

Mutation	Primers
**p.Arg153Pro**	CATCTTGCGCCTCTTTCCCCAGAAAAACAAACGTG<br>CACGTTTGTTTTTCTGGGGAAAGAGGCGCAAGATG
**p.Leu230Pro**	TAGACATGCATTTAAAATGTCCGGTATAGCAGTCTGTATAGCAAG<br>CTTGCTATACAGACTGCTATACCGGACATTTTAAATGCATGTCTA
**p.Arg689Ser**	GAAGCTCACTTCGAAATTCACTCATATCCACAACTATGCTTTG<br>CAAAGCATAGTTGTGGATATGAGTGAATTTCGAAGTGAGCTTC
**p.Arg799Trp**	CTTCACTTCCCCAGACTATGGATTCTCTGGTGC<br>GCACCAGAGAATCCATAGTCTGGGGAAGTGAAG
